# Strength Training Protects High-Fat-Fed Ovariectomized Mice against Insulin Resistance and Hepatic Steatosis

**DOI:** 10.3390/ijms25105066

**Published:** 2024-05-07

**Authors:** Jessica D. M. Santos, José F. T. Silva, Ester dos S. Alves, Alessandra G. Cruz, Anne R. M. Santos, Felipe N. Camargo, Carlos H. Z. Talarico, Carlos A. A. Silva, João Paulo Camporez

**Affiliations:** Department of Physiology, Ribeirao Preto School of Medicine, University of Sao Paulo, Ribeirao Preto 14049-900, Braziljoseturino@usp.br (J.F.T.S.); alvesssester@gmail.com (E.d.S.A.); alessandracruz@usp.br (A.G.C.); annemello1928@hotmail.com (A.R.M.S.); felipe_camargo@usp.br (F.N.C.); carlos.talarico@usp.br (C.H.Z.T.); caguiar@usp.br (C.A.A.S.)

**Keywords:** insulin resistance, menopause, non-alcoholic fatty liver disease

## Abstract

Menopause is characterized by a reduction in sex hormones in women and is associated with metabolic changes, including fatty liver and insulin resistance. Lifestyle changes, including a balanced diet and physical exercise, are necessary to prevent these undesirable changes. Strength training (ST) has been widely used because of the muscle and metabolic benefits it provides. Our study aims to evaluate the effects of ST on hepatic steatosis and insulin resistance in ovariectomized mice fed a high-fat diet (HFD) divided into four groups as follows: simulated sedentary surgery (SHAM-SED), trained simulated surgery (SHAM-EXE), sedentary ovariectomy (OVX-SED), and trained ovariectomy (OVX-EXE). They were fed an HFD for 9 weeks. ST was performed thrice a week. ST efficiently reduced body weight and fat percentage and increased lean mass in OVX mice. Furthermore, ST reduced the accumulation of ectopic hepatic lipids, increased AMPK phosphorylation, and inhibited the de novo lipogenesis pathway. OVX-EXE mice also showed a better glycemic profile, associated with greater insulin sensitivity identified by the euglycemic–hyperinsulinemic clamp, and reduced markers of hepatic oxidative stress compared with sedentary animals. Our data support the idea that ST can be indicated as a non-pharmacological treatment approach to mitigate metabolic changes resulting from menopause.

## 1. Introduction

Menopause, a pivotal phase in a woman’s life, marks the permanent cessation of menstruation after twelve months and a decline in the production of sexual steroid hormones. This transition triggers undesirable symptoms that significantly impact the quality of life [1,2]. In the United States alone, nearly 10 million women are navigating this transformative period [3]. With the life expectancy of women projected to reach 82 years in developed countries by 2025 [4], 30% to 40% of their lives will likely be spent in the postmenopausal phase [5].

During menopause, the combination of physical inactivity, a high-fat diet, and hormonal changes leads to decreased energy expenditure [6,7,8]. This, in turn, triggers metabolic disorders such as increased body fat, obesity, insulin resistance, diabetes mellitus, non-alcoholic fatty liver disease (NAFLD), and cardiovascular diseases [4,9,10,11]. These factors collectively contribute to the development of metabolic syndrome (MetS), which is more prevalent in men than in premenopausal women of similar ages. However, a shift occurs during menopause, making women more susceptible to MetS [1].

Several studies indicate that estrogen has beneficial effects on energy metabolism, and the absence of its action is a preponderant factor in triggering MetS [12,13,14]. The protective effects of this hormone are mediated primarily by estrogen receptor alpha (ERα) [9,13,14,15,16]. The observed energy expenditure reduction during the absence of estrogen action leads to an increase in body weight, especially in the form of an increase in body fat, which is usually associated with a concomitant increase in ectopic fat accumulation, particularly in the liver and skeletal muscle [13,17,18,19]. The excess fat accumulation in the liver is associated with developing hepatic insulin resistance, contributing to the development of NAFLD pathogenesis [1,20]. NAFLD is the most common disease in industrialized countries, characterized by excessive storage of triglycerides (TAGs) in the hepatocyte (above 5%), with its progression characterized by changes such as inflammation and hepatocellular ballooning [18]. Thus, it is possible to observe that menopausal women, probably because of the lack of estrogen action, display an increased prevalence of NAFLD and, consequently, more severe progressions of this disease [1,21]. This progression is also observed in animal models of male ApoE KO mice and ovariectomized female mice [12,22].

The mechanisms underlying the development of NAFLD are of significant importance as they are directly linked to the liver’s energy metabolism imbalance, which includes the conversion of glucose into fatty acids through de novo lipogenesis, and the increased release of fatty acids from adipocytes, leading to the accumulation of hepatic diacylglycerol (DAG) and triglycerides (TAGs) [11,23], phenomena observed in ovariectomized rodents [13], suggesting an important role of estrogen in the development of NAFLD. Furthermore, the transcription factor sterol regulatory element-binding protein 1 (SREBP1) plays a crucial role in activating the enzymes responsible for de novo lipogenesis, with acetyl CoA carboxylase (ACC) being the key enzyme in this process [24], both of which have increased expression in the liver of ovariectomized female mice fed with an HFD [13]. This heightened lipid synthesis can trigger hepatic lipotoxicity and disrupt the electron transport chain, producing reactive oxygen species (ROS) and subsequent oxidative stress [24]. These mechanisms are pivotal in the development and progression of NAFLD, which is observed in both experimental models of menopause and postmenopausal women [1,13,14].

Considering the complex nature of NAFLD, lifestyle interventions such as a balanced diet and physical exercise have proven to be effective alternatives for its prevention and treatment. Physical exercise, in particular, stands out as a non-pharmacological therapeutic option for weight reduction, improvement of fatty liver, and insulin resistance [25,26]. The benefits of physical exercise extend beyond these effects, providing practitioners with a considerable number of important physiological and metabolic adaptations, especially in special populations. For instance, strength training (ST) can significantly improve muscular strength when tailored to the individual’s load, volume, and intensity [27]. Other studies using rodents as an experimental model have also demonstrated the positive impact of chronic ST on body weight, adipocyte area, and inflammatory markers [28,29], as well as its ability to modulate molecular pathways related to glucose tolerance, insulin sensitivity, and lipid metabolism in the liver and skeletal muscle [29]. Short-term ST has been found to reduce hepatic fat accumulation and enhance liver insulin sensitivity in male rodents [30]. A recent study by Nunes et al. [31] further supports these findings, showing that high-volume resistance training can improve the metabolic profile and inflammation in obese and postmenopausal women.

However, there is still a limited amount of evidence on the effects of ST and the metabolic changes associated with menopause since most articles emphasize other training methods. Since the ovariectomized mouse model is established in the literature as a way to mimic human menopause [32], our study aims to evaluate the effects of ST on hepatic lipid metabolism and insulin resistance in ovariectomized mice fed an HFD. We hypothesize that ST can prevent the metabolic changes triggered by menopause.

## 2. Results

### 2.1. Maximal Load Capacity

**Figure 1:**
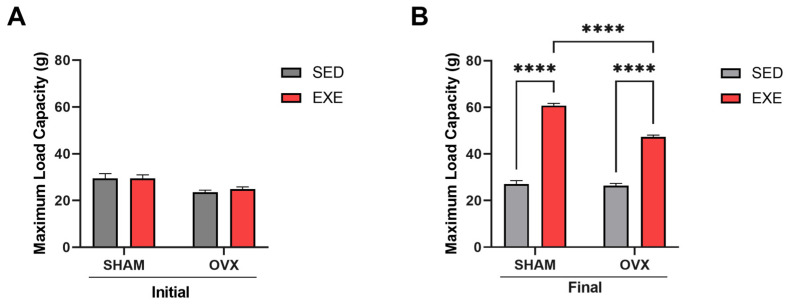
Strength training increases muscle strength after 8 weeks of exercise. Analysis of the maximum load test performed before and after eight weeks of training and after eight weeks. (**A**) Maximum initial load capacity of the SHAM (SED and EXE) and OVX (SED and EXE) groups. (**B**) Maximum final load capacity of the SHAM (SED and EXE) and OVX (SED and EXE) groups. Data are represented as means ± SEM (*n* = 8), with differences considered significant when *p* < 0.05. Analysis was performed with two-way ANOVA with Tukey’s post hoc test. **** *p* < 0.0001.

The maximum load test (MLC) was performed to evaluate the animals’ maximum strength before and after training. We observed that there were no significant differences between the sedentary SHAM and OVX groups in pre-training (Figure 1A). After 8 weeks of training, we could identify that there was an increase in muscle strength capacity in the SHAM-EXE and OVX-EXE groups when compared with the sedentary groups, respectively (Figure 1B), showing the effectiveness of the training in gaining muscle strength. It is worth noting that between the trained groups, there were also significant differences in muscle strength, with SHAM-EXE showing greater muscle strength when compared with OVX-EXE, indicating that ovariectomy can affect the gain in muscle strength because of estrogen deficiency (Figure 1B).

### 2.2. Body Composition and Caloric Intake of Mice after 8 Weeks of ST

**Figure 2:**
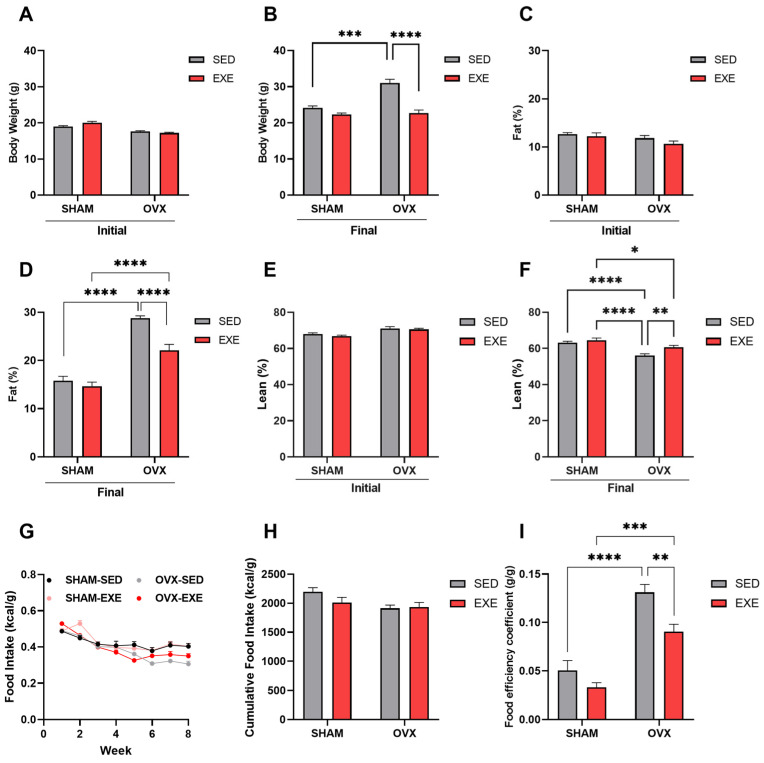
Strength training effects on the body composition of animals. Analysis of the body composition of ovariectomized and non-ovariectomized animals submitted to strength training for eight weeks. (**A**) Initial body weight of the SHAM (SED and EXE) and OVX (SED and EXE) groups. (**B**) Final body weight of the SHAM (SED and EXE) and OVX (SED and EXE) groups. (**C**) Initial percentage of body fat in the SHAM (SED and EXE) and OVX (SED and EXE) groups. (**D**) Final percentage of body fat in the SHAM (SED and EXE) and OVX (SED and EXE) groups. (**E**) Initial percentage of lean mass in the SHAM (SED and EXE) and OVX (SED and EXE) groups. (**F**) Final percentage of lean mass in the SHAM (SED and EXE) and OVX (SED and EXE) groups. (**G**) Graph of food intake in the SHAM (SED and EXE) and OVX (SED and EXE) groups after 8 weeks of HFD. (**H**) Cumulative food intake. (**I**) Food efficiency coefficient. Data are represented as mean ± SEM (n = 7–8 per group), with differences considered significant when *p* < 0.05. Analysis was carried out using two-way ANOVA with Tukey’s post hoc test. (* *p* < 0.05); (** *p* < 0.01); (*** *p* < 0.001); (**** *p* < 0.0001).

Analyzing the body weight of the mice, we observed that after 8 weeks of HFD, the animals in the OVX-SED group showed a ~28% increase in body weight when compared with the SHAM-SED group (Figure 2B). Subsequently, at the end of the 8 weeks of training, we observed that the animals in the SHAM-EXE and OVX-EXE groups showed a reduction in body weight of ~8% and ~26% when compared with the sedentary groups, respectively. The SHAM-SED and SHAM-EXE groups showed no significant differences in body weight. These data demonstrated that ST effectively prevented body weight gain.

Associated with the increase in body weight, the OVX-SED animals showed an ~82% increase in total body fat (Figure 2D) and a reduction in lean mass when compared with the animals in the SHAM-SED group (Figure 2F). The OVX-EXE rats had a ~36% reduction in body fat compared with the OVX-SED rats, thus showing that strength training was effective in attenuating total body fat gain (Figure 2D). In addition, there was a gain in lean mass in the OVX-EXE animals when compared with the OVX-SED animals, indicating that exercise training was practical in gaining lean mass after ovariectomy (Figure 2F). It is worth noting that there was also a significant difference in the percentage of body fat and lean mass between the OVX-EXE and SHAM-EXE groups, showing that SHAM-EXE obtained a reduction in the percentage of fat and an increase in lean mass when compared with OVX-EXE, which may indicate that the absence of estrogen influences the gain of lean mass (Figure 2D–F). The SHAM-SED and SHAM-EXE groups showed no significant difference in fat percentage.

In addition, during the 9 weeks of intervention, food intake was monitored in all groups. We observed no significant differences in the cumulative caloric intake over the weeks between the OVX-SED and OVX-EXE, SHAM-SED, and SHAM-EXE groups (Figure 2G,H). With this, OVX-SED mice displayed a higher food efficiency coefficient compared with the SHAM mice (Figure 2I), while OVX-EXE mice presented a reduced food efficiency coefficient (Figure 2I). Thus, with this data set, we demonstrate that strength training, performed three times a week, attenuates weight gain, prevents the storage of body fat induced by ovariectomy, and induces an increase in lean mass, regardless of estrogen.

### 2.3. ST Improves Glucose Tolerance and Insulin Sensitivity in Ovariectomized Mice

**Figure 3:**
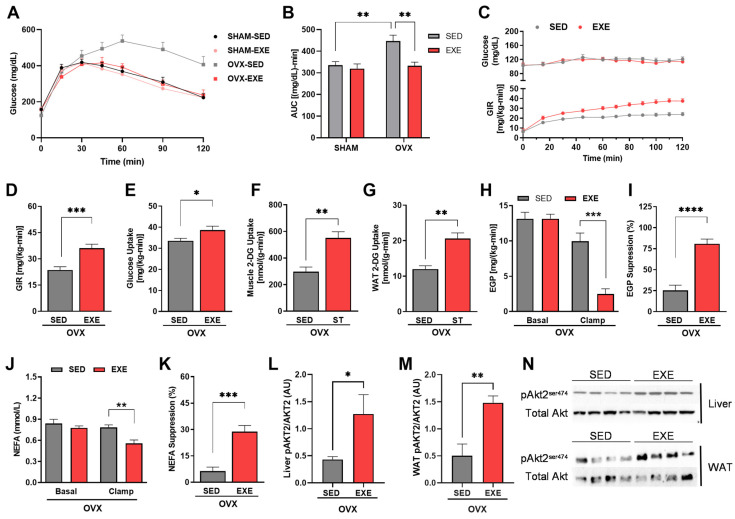
Strength training increases glucose tolerance and reduces insulin resistance in ovariectomized female mice. Analysis of glucose tolerance and insulin sensitivity. (**A**) Blood glucose levels during the GTT in the SHAM (SED and EXE) and OVX (SED and EXE) groups. (**B**) Area under the curve (A.U) of blood glucose levels in the SHAM (SED and EXE) and OVX (SED and EXE) groups. (**C**) Glucose levels and glucose infusion rate during the euglycemic–hyperinsulinemic clamp in the OVX groups (SED and EXE). (**D**) Average of the last 40 min of the glucose infusion rate in the OVX groups (SED and EXE). (**E**) Insulin-stimulated whole-body glucose uptake in the OVX groups (SED and EXE). (**F**) 2-deoxyglucose uptake in muscle tissue during the hyperinsulinemic–euglycemic clamp. (**G**) 2-deoxyglucose uptake in white adipose tissue during the hyperinsulinemic–euglycemic clamp. (**H**) Basal endogenous glucose production and during the euglycemic–hyperinsulinemic clamp in the OVX groups (SED and EXE). (**I**) Suppression of endogenous glucose production during the euglycemic–hyperinsulinemic clamp. (**J**) Non-esterified fatty acids (NEFA) basal and during the euglycemic–hyperinsulinemic clamp. (**K**) Suppression (%) of non-esterified fatty acids. (**L**) Quantification of AKT2 phosphorylation in the liver. (**M**) Quantification of AKT2 phosphorylation in the WAT. (**N**) Western blot image of AKT2 phosphorylation in the liver and WAT. Data are represented as means ± SEMs (n = 7–10), with differences considered significant when *p* < 0.05. In (**A**–**C**), the analysis was performed using two-way ANOVA with Tukey’s post hoc test. For (**D**–**L**), a *t*-test was performed. Significance levels are *p* < 0.05. (* *p* < 0.05); (** *p* < 0.01); (*** *p* < 0.001); (**** *p* < 0.0001).

Our study’s findings regarding glucose tolerance are significant. We observed that the glucose concentration remained high in the animals in the OVX-SED group (Figure 3A), as well as in the AUC group (Figure 3B) during the GTT, when compared with the other groups of animals, showing greater glucose intolerance in OVX mice. However, the OVX-EXE animals had a reduction in glucose concentration during the GTT (Figure 3A), showing a decreased AUC (Figure 3B) when compared with the OVX-SED animals (*p* < 0.001), demonstrating that ST performed for eight weeks enabled greater glucose tolerance. Furthermore, it is worth noting that the mice in the OVX-EXE group presented glucose concentrations similar to the SHAM-EXE group, and in AUC, there were no significant differences during the GTT time, showing that ST is effective in attenuating the glycemic profile, regardless of the hormonal changes triggered by ovariectomy. There were no differences between the SHAM-SED and SHAM-EXE groups.

The implications of our study’s results regarding insulin sensitiity are profound. During the clamp, we demonstrated that the rate of glucose infusion to maintain euglycemia was significantly higher in the EXE animals than in the SED animals (Figure 3C,D), showing that the trained animals had greater insulin sensitivity. In addition, we showed that the EXE group had greater glucose uptake throughout the body when compared with the SED animals (Figure 3E), as well as increased glucose uptake in muscle tissue and white adipose tissue (Figure 3F,G). Furthermore, the trained mice showed greater suppression of insulin-stimulated endogenous glucose production compared with the SED animals (Figure 3H,I), suggesting greater hepatic sensitivity to insulin. Moreover, the trained animals had greater insulin-stimulated NEFA suppression (Figure 3J,K), demonstrated by reduced plasma NEFA concentrations during the clamp. Additionally, we showed that the EXE animals presented greater hepatic and adipose tissue insulin-stimulated AKT2 phosphorylation (Figure 3L–N) when compared with the sedentary animals, suggesting higher insulin sensitivity in liver and adipose tissue.

### 2.4. Lipid Profile and Oxidative Stress in the Liver after 8 Weeks of ST

**Figure 4:**
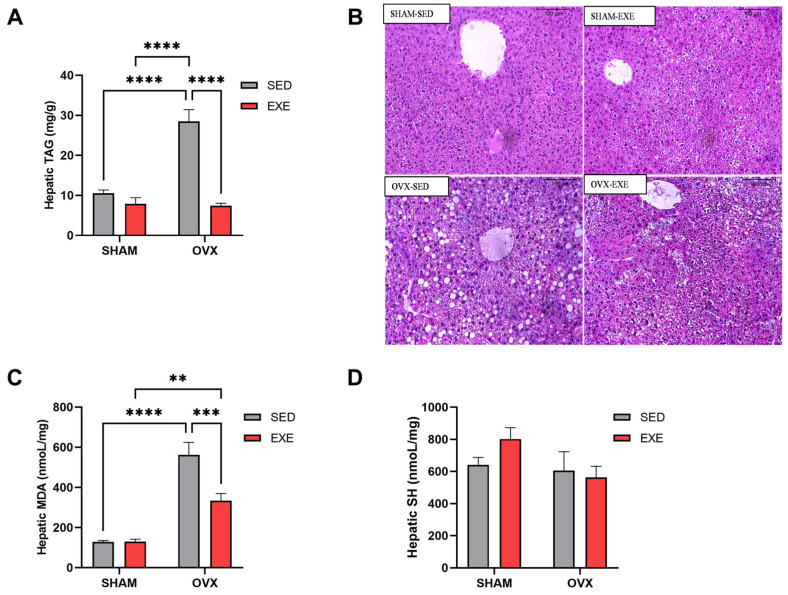
Strength training effects on lipid content and oxidative stress markers. Statistical analysis of lipid content and oxidative stress markers in female mice fed an HD, whether or not they were subjected to strength training. (**A**) Hepatic triglyceride levels in the SHAM (SED and EXE) and OVX (SED and EXE) groups. (**B**) Histological analysis of the liver in HE staining of the SHAM (SED and EXE) and OVX (SED and EXE) groups, 100 µm. (**C**) Hepatic MDA concentration in the SHAM (SED and EXE) and OVX (SED and EXE) groups. (**D**) SH levels in the liver in the SHAM (SED and EXE) and OVX (SED and EXE) groups. Data are represented as means ± SEMs (n = 7–8 per group), with differences considered significant when *p* < 0.05. Analysis was performed with two-way ANOVA with Tukey’s post hoc test. (** *p* < 0.01); (*** *p* < 0.001); (**** *p* < 0.0001).

OVX-SED mice achieved more significant hepatic TAG accumulation after 8 weeks of HFD when compared with SHAM-SED mice, demonstrating that the absence of estradiol potentiates TAG accumulation in the liver (Figure 4A). Interestingly, the OVX-EXE group completely reversed the increase in hepatic TAG concentration when compared with the OVX-SED group, indicating the efficiency of ST in reducing TAG accumulation in the liver (Figure 4A). There was no significant difference between the SHAM groups (Figure 4A).

Furthermore, we evaluated malonaldehyde (MDA) levels in liver tissue, an indirect marker of lipid peroxidation. We observed that the OVX-SED group showed an increase in MDA concentrations compared with the SHAM-SED group. In contrast, the OVX-EXE group had reduced MDA concentrations when compared with the OVX-SED group, demonstrating the effects of ST in reducing indirect indicators of oxidative stress in liver tissue (Figure 4C). We also measured the sulfhydryl group (SH) in the liver, a marker of antioxidant action, and no significant difference was observed in any of the groups (Figure 4D).

### 2.5. Effects of ST on Hepatic Lipid Metabolism Pathways

**Figure 5:**
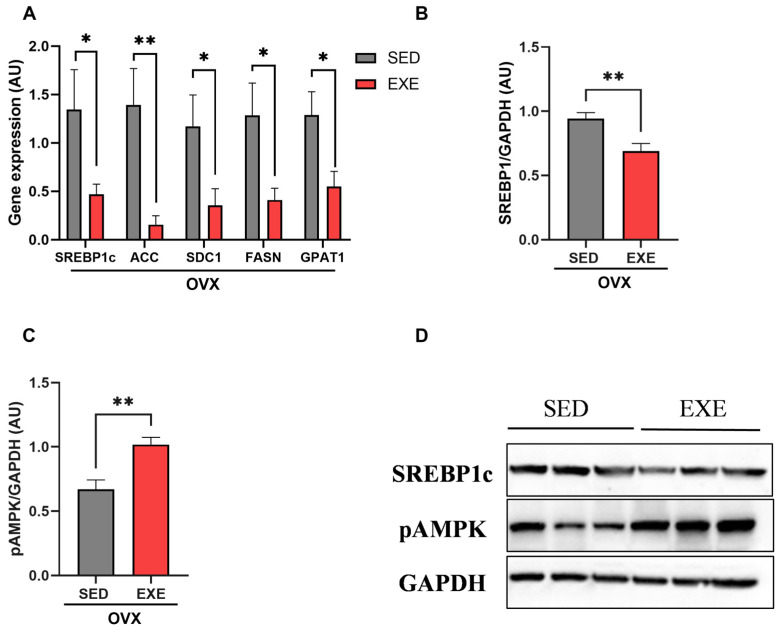
Gene and protein expression of molecules involved in the process of de novo lipogenesis and lipid oxidation in the liver. Analysis of genes and proteins related to lipid metabolism in the liver. (**A**) Gene expression of molecules involved in de novo lipogenesis including SREBF1, ACC, SCD1, FASn, and GPAT1 in the OVX groups (SED and EXE). (**B**) Quantification of SREBP1c protein expression. (**C**) Quantification of pAMPK protein expression. (**D**) Western blot image of SREBP1c protein and AMPK phosphorylation. Data are represented as means ± SEMs (n = 5–7 per group), with differences considered significant when *p* <0.05. Analysis was performed with a *t*-test. (* *p* < 0.05); (** *p* < 0.01).

After verifying the results related to ectopic lipid content in the liver, we investigated genes and proteins associated with lipid synthesis and oxidation only in the groups of ovariectomized mice. The gene expression of the hepatic lipogenic transcription factor (*SREBP1c*) and other genes downstream of the de novo lipogenesis pathway, such as acetyl CoA carboxylase (*ACC*), stearoyl-CoA desaturase 1 (*SCD1*), fatty acid synthase (*FASn*), and glycerol-3-phosphate acyltransferase (*GPAT1*), was significantly lower in the trained mice than in the sedentary animals (Figure 5A). Furthermore, the expression of the SREBP1c protein was reduced in the EXE groups when compared with SED (Figure 5B,D), showing that training suppressed the activation of this pathway. The phosphorylation of the AMPK protein, related to hepatic lipid oxidation, was increased in the trained groups compared with the sedentary ones (Figure 5C,D).

### 2.6. ST in the Gene and Protein Expression of Cytokines in the Liver of Ovariectomized Mice

**Figure 6:**
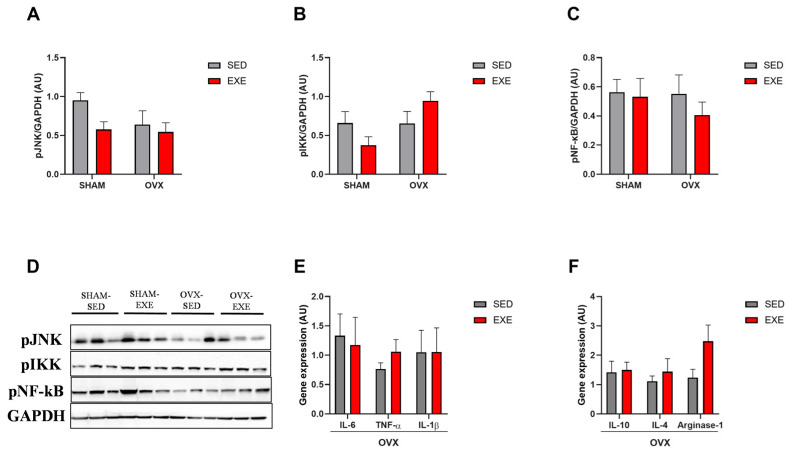
Strength training effects on cytokines gene expression. Analysis of gene expression of pro- and anti-inflammatory cytokines in the liver. (**A**) Quantification of protein expression of JNK phosphorylation in the SHAM (SED and EXE) and OVX (SED and EXE) groups. (**B**) Quantification of protein expression of IKK phosphorylation in the SHAM (SED and EXE) and OVX (SED and EXE) groups. (**C**) Quantification of the protein expression of NF-kB phosphorylation in the SHAM (SED and EXE) and OVX (SED and EXE) groups. (**D**) Western blot image of protein phosphorylation JNK, IKK, and NF-kB. (**E**) Anti-inflammatory cytokine genes IL-10, IL-4, and Arginase-1 in the OVX groups (SED and EXE). (**F**) Pro-inflammatory cytokine genes IL-6, TNF-α, and IL1-β in the OVX groups (SED and EXE). Data are represented as means ± SEMs (n = 6–8 per group), with differences considered significant when *p* < 0.05. Analysis was performed with two-way ANOVA with Tukey’s post hoc test.

Since ectopic lipid accumulation is associated with activating various inflammatory signaling pathways, we evaluated the protein expression of molecules related to activating pro-inflammatory genes. The phosphorylation of IκB Kinase (IKK), c-Jun N-terminal Kinase (JNK), and Nuclear Factor kappa B (NF-kb) showed no significant differences in the SHAM (SED and EXE) and OVX (SED and EXE) groups (Figure 6A–D). Subsequently, we analyzed the gene expression of genes related to the pro- and anti-inflammatory pathways, specifically in the OVX groups. *IL-6*, *IL1-β*, and *TNF-α* genes did not show significant differences in the trained and sedentary groups (Figure 6E). In addition, no significant differences were found in the gene expression of the cytokines *IL-10*, *IL-4*, and arginase-1 (Figure 6F). From this perspective, we can demonstrate that after ovariectomy and 8 weeks of HFD, there was no hepatic inflammatory process or any effect of strength training on these parameters.

## 3. Discussion

In the present study, we showed that ST attenuates body weight gain associated with a lack of estrogen and a high-fat diet. It is possible that these effects are, at least partially, due to the induction of a negative energy balance by the training since the animals did not show changes in eating behavior, but exercise would evidently be capable of leading to greater energy expenditure. With this, it was possible to observe that ST led to a reduction in the body fat percentage and ectopic lipid accumulation in the liver, which improved insulin sensitivity and glucose tolerance in the OVX mice.

During menopause, there is an accelerated decline in muscle function, mass, and strength in women [33] because of the reduction in the production of estrogen. This hormone contributes significantly to maintaining muscle mass [34] and is associated with aging [35]. Therefore, reduced MLC indicates loss of muscle strength after ovariectomy in female mice fed an HFD, corroborating a previous study [36]. Considering that ST is an efficient approach to preventing loss of muscle strength during aging [37], we used stair climbing to mimic ST performed in humans [38]. This training model in our study proved to be effective by increasing muscle strength in the OVX mice equally to the SHAM animals (Figure 1), indicating that this type of training contributes to improving muscular performance independently of the hormonal action of estrogen. Other studies with resistance training, moderate loads, and high repetitions also showed increased muscle strength in OVX mice [39].

Furthermore, during menopause, there is also a reduction in total and resting energy expenditure, resulting in changes in energy balance [40], which subsequently leads to increased body weight and abdominal adiposity excess in both human and animal models [9,13,22,41]. These data are similar to ours, where the OVX mice showed increased weight and percentage of total body fat. However, ST proved to be effective in mitigating the increase in weight and body fat percentage (Figure 2). Interestingly, these weight and body fat results were independent of eating behavior since the trained animals consumed the same energy during the training period as the sedentary animals (Figure 2). Considering that physical exercise increases energy expenditure [42], it is possible to speculate that the trained OVX animals presented a negative energy balance, leading to the protective effects of training on weight and fat gain. These data corroborate other studies that also showed a reduction in body weight [36,43] and adiposity [44] after physical training sessions with OVX rodents.

Excess body fat is associated with other comorbidities, mainly glucose intolerance and insulin resistance [1]. We observed that the HFD-fed OVX mice had greater glucose intolerance than the SHAM animals (Figure 3). Other studies corroborate these findings, showing that the absence of estrogen promotes glucose intolerance in male and ovariectomized female mice, confirming the importance of this hormone in glycemic control [13,14,15]. However, ST performed three times a week allowed for an improvement in glycemic metabolism in the OVX animals, regardless of estrogen. In line with our findings, Costa et al. [45] showed that ST performed in obese male rats improved glucose tolerance.

Also, ST effectively improves insulin sensitivity, as demonstrated by the euglycemic–hyperinsulinemic clamp (Figure 3). Experimental and clinical studies have shown that other types of training (aerobic, HIIT, resistance) or their combination also promote greater insulin sensitivity in both men and women [46,47]. Thus, we demonstrated that the ST method protected the HFD-fed female OVX mice from insulin resistance by increasing peripheral and hepatic insulin sensitivity (Figure 3).

The experimental model of menopause used in this study, i.e., ovariectomized female mice, has been associated with the development of hepatic steatosis connected with hepatic insulin resistance, generally associated with the consumption of an HFD [1,13]. Interestingly, previous studies have shown that physical training reduces the accumulation of intrahepatic lipids, whether associated with weight loss [48], mobilizing the use of hepatic TAG, reducing tissue accumulation, and preventing steatosis [49] or not. Our data demonstrated that the ST protocol used in this study prevented hepatic fat accumulation in the OVX mice (Figure 4), regardless of the presence of estrogen. Furthermore, the reduction in hepatic TAG caused by ST may have directly impacted the improvement in hepatic insulin sensitivity. Increased hepatic TAG content has been directly associated with an increased content of DAG [15,50,51,52,53], a signaling lipid species. Hepatic DAG leads to the activation of one of the new isoforms of PKC, PKCε, which, when activated in the hepatocyte, phosphorylates the insulin receptor at Threonine1160 [54,55], leading to the inactivation of this receptor, as it makes its autophosphorylation on tyrosine impossible. This phenomenon has been observed also in HFD-fed OVX mice [13]. Therefore, we can suggest that ST promoted an improvement in hepatic sensitivity to insulin by reducing the accumulation of TAG in the liver of the OVX animals.

Impairment of lipid metabolism and insulin resistance leads to the inhibition of glycogen synthesis enzymes and redirects glucose to the lipogenic pathway, stimulating SREBP1c and its downstream targets and increasing intrahepatic TAG production [11]. However, we observed that ST efficiently inhibited SREBP1c and all downstream enzymes of the lipogenic pathway (Figure 5). Also, ST led to an increase in AMPK phosphorylation (Figure 5), which is associated with an increase in its activity, suggesting that fatty acid oxidation may be increased in the hepatocytes of the trained animals, which would help to prevent hepatic steatosis. In addition, this enzyme is also responsible for inhibiting hepatic glucose production. Studies have already shown that other types of training have positively regulated the AMPK pathway [46] and have indicated that short-term exercise training reduced hepatic lipogenesis [30]. Another study with OVX mice also found that resistance training prevented hepatic steatosis by altering the gene expression of molecules related to hepatic lipid metabolism [56]. Thus, the beneficial effects of exercise training performed three times a week, preventing hepatic steatosis and insulin resistance, may be associated with reduced de novo lipogenesis and increased hepatic fat oxidation.

In addition to lipid accumulation being a significant factor in insulin resistance, there is also an increase in the production of reactive oxygen species (ROS), culminating in a state of oxidative stress, inducing injury and inflammation in the hepatocyte, and causing liver disease progression. We saw that the OVX animals had more significant hepatic oxidative stress compared with the SHAM mice (Figure 4). Our study demonstrates that ST is an effective intervention in reducing oxidative stress induced by ovariectomy (Figure 4). A previous study with other training methods showed that exercise improves the hepatic oxidative state in rodents [57]. It is worth noting that exercises can be beneficial in mitigating oxidative damage, depending on the volume and intensity established [58], with the protocol used in our work being effective in reducing hepatic oxidative stress.

Concatenating our findings, ST performed three times a week promoted beneficial effects in attenuating weight gain, the percentage of body fat associated with consuming a high-fat diet, and the absence of estrogen in females. Furthermore, it reduced hepatic TAG accumulation and oxidative stress, improving glucose tolerance and insulin sensitivity. Taken together, our data show that ST may be an alternative approach to pharmacological treatment and hormone replacement to attenuate metabolic changes resulting from menopause.

## 4. Materials and Methods

### 4.1. Animals

**Figure 7:**
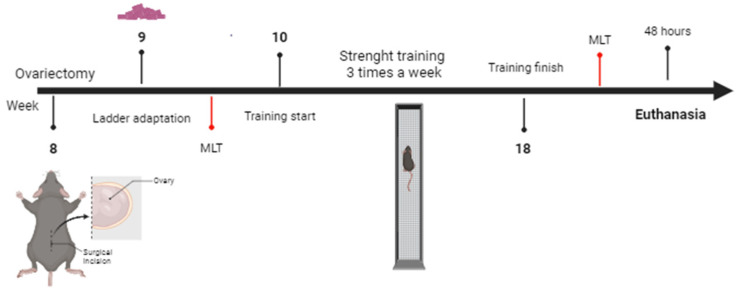
Experimental Design. Created in Biorender.

Female *C57BL/6J* mice, 8 weeks old, were used and maintained in a temperature-controlled room at 22 ± 2 °C with ad libitum access to food and water, subjected to a 12-h light–dark cycle (light from 6 a.m. to 6 p.m.). The mice were divided into four groups (n = 8) as follows: sedentary sham surgery (SHAM-SED); trained sham surgery (SHAM-EXE); sedentary ovariectomy (OVX-SED); and trained ovariectomy (OVX-EXE). These animals were fed a high-fat diet (HFD) containing 45% calories from fat (D12451, Research Diets, NJ, USA) for 9 weeks. All experiments carried out were previously approved in accordance with the guidelines of the Ethics Committee of Ribeirao Preto School of Medicine of the University of São Paulo, with protocol 1096/2022. No exclusion criteria were used, and all researchers were aware of the group allocation at the different stages of the experiment. The experimental design is illustrated in Figure 7.

### 4.2. Ovariectomy

Bilateral ovariectomy was performed in females at 8 weeks of age. After being anesthetized with isoflurane, their hair was removed from the surgical site and prepared with an appropriate skin disinfectant (Nolvasan^®^ followed by alcohol). Then, a small dorsal incision was made, and the ovaries were clamped to stop the bleeding and subsequently removed. Then, suturing was performed, and the animal was kept in a warm and clean cage for recovery, with carprofen administered.

### 4.3. Ladder Adaptation

The strength training was performed with a specific ladder for rats, with a height of 1110 mm, a base of 300 × 350 mm, a distance between steps of 6 mm, and an inclination of 80°. Before starting strength training, the animals underwent an adaptation period, as previously published [59], where all rats climbed the stairs for 5 consecutive days without load. The familiarization of the animals consisted of climbing 10 times in a row without interruptions, in 3 series with pauses and 2 min of passive rest. The animals were placed at the base of the ladder and induced to climb to the top of the ladder. This adaptation aimed to familiarize them with the protocol, thus reducing the animals’ stress.

### 4.4. Maximum Load Capacity (MLC)

After familiarization with the ladder, MLC was performed in order to determine the initial training load. The test began with a climb carrying a cylindrical apparatus fixed to the proximal portion of each animal’s tail with fasteners, with an initial load of 60% of the animal’s body weight. After completion, an incremental load of 5% of each animal’s body weight was added to the charging device. This procedure was repeated successively, with a 1-min rest interval between climbs until a load was reached with which the animal could not climb the entire length of the ladder, thus characterizing a failure. The largest load transported was considered [59,60]. This test was also carried out on the 4th week of training to adjust the load and at the end of training to evaluate the maximum load that the animal was able to carry, characterizing a failure. The largest load transported was considered [59].

### 4.5. Strength Training Protocol (ST)

The training protocol was carried out 3 times a week for 8 weeks, with adjustments in the intensity and volume of the exercise according to the loads identified in the MCL, with a 2-min rest between each series performed, following the protocol by Luciano et al. [61] with adaptations (Table 1).

### 4.6. Glucose Tolerance Test

After 6 h of food restriction, glucose (2 g/kg body weight, 20% dextrose) was injected into the mice intraperitoneally. Blood samples for measuring glucose were taken by tail bleeding 0, 15, 30, 45, 60, 90, and 120 min after injection.

### 4.7. Magnetic Resonance

Body composition was assessed with a whole-body analyzer (Bruker’s minispec Whole Body Composition Analyzer) based on TD-NMR for the accurate analysis of the measurement of lean mass, body fluid, and fat. All animals were placed in a cylindrical containment tube and inserted into the device for a maximum period of 60 to 80 s.

### 4.8. Tissue Lipid Content

After 6 h of food restriction, the animals were euthanized, and the liver tissue was removed for analysis of lipid content. Tissue triglycerides (TAGs) were extracted using the Bligh and Dyer method [62] and measured using TAG reagent (Bioclin^®^).

### 4.9. In Vivo Assessment of Insulin Sensitivity

To assess insulin sensitivity in vivo, a normoglycemic–hyperinsulinemic clamp was performed, as previously described [51,53], 48 h after the last training session. A jugular vein catheter was implanted 5 days before the normoglycemic–hyperinsulinemic clamp. To measure basal endogenous glucose production (EGP), [3-3H]-glucose (Perkin-Elmer Life Sciences, Waltham, MA, USA) was infused at a rate of 0.05 μCi/min for 120 min after 6 h of food restriction. After this basal infusion, the normoglycemic–hyperinsulinemic clamp was conducted in awake animals for 120 min with an initial infusion of insulin for 3 min (29 mU/kg) followed by a continuous infusion of 3 mU/kg.min-1 of human insulin (Novolin; Novo Nordisk, Bagsværd, Denmark). A continuous infusion of [3-3H]-glucose (0.1 μCi/min) and a variable infusion of dextrose (20%) were also performed to maintain normal blood glucose (~120 mg/dL). After 75 min of the start of the clamp, a bolus of 10µCi of 2-deoxy-d-[1-14C] glucose (PerkinElmer) was injected to estimate insulin-stimulated tissue glucose uptake. Plasma samples were collected via the carotid artery at 0, 15, 30, 45, 60, 70, 80, 90, 100, 110, and 120 min [45]. Furthermore, the animals received a solution containing albumin intravenously, artificially mimicking plasma during the insulin infusion period, to compensate for the volume of blood removed from plasma samples. At the end of the clamp, the animals were anesthetized with an injection of sodium pentobarbitol (150 mg/kg), and the peri-uterine adipose tissue, skeletal muscle (gastrocnemius), and livers were removed, immediately frozen in liquid nitrogen, and stored at −80 °C for subsequent analyses.

### 4.10. Western Blot

The liver was homogenized in RIPA buffer at 4 °C (1% Triton-X-100, 100 mM Tris (pH 7.4, 100 mM sodium pyrophosphate, 100 mM sodium fluoride, 10 mM EDTA, 10 mM sodium orthovanadate and PMSF 2 mM) with Polytron PTA 20S homogenizer (Brinkmann Instruments, Riverview, FL, USA). Tissue extracts were centrifuged at 12,000 rpm at 4 °C for 20 min to remove insoluble material. After centrifugation, the total protein content was quantified using the Bradford method (BioRad, Hercules, CA, USA). Samples were treated with Laemmli buffer containing 200 mM DTT and 30 mg of total proteins solubilized from mouse liver fragments. These samples were subjected to polyacrylamide gel electrophoresis.

The proteins separated in the gel were transferred electrically to a PVDF membrane using a semi-dry transfer system (Bio-Rad) for 120 min. PVDF membranes were incubated with a blocking solution (5% Molico^®^ skimmed milk, 10 mM Tris, 150 mM NaCl, and 0.02% Tween 20) at 4 °C for 2 h to reduce nonspecific binding of proteins to the membranes. Subsequently, the membranes were incubated with the following antibodies: SREBP1 (Santa Cruz Biotechnology, Inc., Santa Cruz, CA, USA), phospho-AMPK (Santa Cruz Biotechnology, Inc., Santa Cruz, CA, USA), phospho-IKK (Cell Signaling Technology, Inc., Danvers, MA, USA), phospho-NFkappaB (Cell Signaling Technology, Inc., Danvers, MA, USA), phospho-JNK (Cell Signaling Technology, Inc., Danvers, MA, USA) phospho-AKT2ser474 (Cell Signaling Technology, Inc., Danvers, MA, USA), and total AKT (Cell Signaling Technology, Inc., Danvers, MA, USA). All membranes were incubated with GAPDH (Santa Cruz Biotechnology, Inc., Santa Cruz, CA, USA) to control the amount of protein in the membrane.

These incubations were carried out with a blocking solution (3% BSA instead of milk) for 12 h at 4 °C, and the concentration of each antibody was between 1:200 and 1:1000. Then, they were washed with the blocking solution without milk or BSA for 30 min. After that, the membranes were incubated with the second antibody, conjugated to peroxidase, for 2 h at room temperature and, shortly after, with the solution for chemiluminescence detection, as described in the commercial kit protocol (ECLPlus, Amersham, UK). Light emission was detected and visualized using radiographic films. The intensity of the bands was quantified by optical densitometry using a band intensity analysis program (Scion Image Software 4.0, Scion Corporation, Frederick, MD, USA).

### 4.11. Assessment of Gene Expression—Polymerase Chain Reaction (PCR)

The livers were removed, and 50 mg of a sample was homogenized in 1 mL of Trizol^®^ (Life Technologies—Carlsbad, CA, USA) for mRNA extraction. The RNA concentration reading was evaluated at 260 nm and purity from the 260/280 nm ratio in the nanodrop device (Thermo Scientific™—Waltham, MA, USA). Then, cDNA was prepared through the reverse transcription reaction (High-Capacityc DNA kit, Applied Biosystems—Waltham, MA, USA). Gene expression was analyzed by real-time PCR (Rotor Gene Q—Qiagen, Hilden, Germany) and SYBR Green fluorescent probe (Platinum^®^ SYBR^®^ Green qPCR Supermix UDG, Invitrogen—Waltham, MA, USA).

### 4.12. Analysis of Oxidative Stress Markers

Lipid oxidation was determined by measuring thiobarbituric acid reactive substances (TBARSs) according to the method described in [63] with some modifications as follows: 200 µL of homogenate, 350 µL of 20% acetic acid (pH 3.5), and 600 µL of thiobarbituric acid (TBA—0.5%, dissolved in acetic acid). The tubes were incubated in a thermostated bath for 1 h at 85 °C. Then, after cooling in an ice bath, 50 µL of 8.1% SDS was added and centrifuged at 10,000 rpm for 15 min at 4 °C, and the absorbance was measured at 532 nm.

Sulfhydryl groups (SH) were quantified to identify the antioxidant level of liver tissue. Aliquots of 50 µL of liver sample were mixed in 300 µL of Tris-EDTA buffer, pH 8.2. Then, the first reading (A) was performed on a plate reader at 412 nm. After reading, the samples were mixed with 10 µL of 10 mM DTNB diluted in methanol (4 mg/mL) and rested in the dark. At the end of 15 min, the second absorbance reading (A2) was taken. The SH concentration was calculated according to the following equation: (A2 − A1) − B × 1.57 mM × 1000. The result is expressed in nmol/mL of tissue.

### 4.13. Liver Morphology

To evaluate liver morphology, each liver was fixed in 10% formaldehyde solution for 8 h, in individual cassettes. Subsequently, the fixed samples were kept overnight in 70% alcohol. The samples were then dehydrated through a series of baths in 95% alcohol, 100% alcohol, and xylene. Once the samples were dehydrated, the tissue samples were embedded in paraffin at 60 °C. A microtome (Zeiss, Jena, Germany) was used to cut the samples into 5-micron slices. The slices were stained with hematoxylin and eosin (H&E), the cellular morphology was evaluated, and the intracellular vacuoles were quantified. Twenty images from each animal were obtained using a Nikon Eclipse Ti-U microscope at 20× magnification coupled with a Nikon DS-R1 digital camera and NIS-Elements BR 3.1 software. These images were projected onto a high-resolution LCD monitor.

### 4.14. Statistics

The results were analyzed using GraphPad Prism version 9.0 (GraphPad Software, La Jolla, CA, USA). The sample distribution was performed using the Kolmogorov–Smirnov normality test. The results were expressed as means ± SEMs. Statistical analyses were performed using two-way analysis of variance (ANOVA) followed by Tukey’s test for homogeneity of variances, and, when necessary, the *t*-test for unpaired samples was used. The minimum acceptable significance level was *p* < 0.05. There were no exclusions of any experimental group, experimental units, or data points.

## Figures and Tables

**Table 1:** Strength training protocol, consisting of changes in training volume and intensity, starting with higher repetitions and moderate load and progressing to increasing load and reducing volume.

Week	Intensity (% Maximal Load)	Volume
1	60%	3/12
2	80%	4/8
3–4	100%	5/6
5–6	120%	6/5
7–8	140%	6/4

## Data Availability

The data presented in this study are available on request from the corresponding author.
